# What to spot and what not to miss: key anomalies during the developing dentition

**DOI:** 10.1038/s41415-025-9114-4

**Published:** 2025-12-19

**Authors:** Nusaybah Elsherif, Vignesh Eswara Murthy, Howard Moseley

**Affiliations:** 239822928888911554904 Department of Orthodontics, Watford General Hospital and Department of Orthodontics, Eastman Dental Institute, UCL, London, United Kingdom; 047094965028761510868 Department of Orthodontics, Royal National ENT and Eastman Dental Hospitals, University College London Hospitals NHS Foundation Trust, London, WC1E 6DG, United Kingdom; 021898775386578410975 Department of Orthodontics, Watford General Hospital and Royal National ENT and Eastman Dental Hospitals, University College London Hospitals NHS Foundation Trust, United Kingdom

## Abstract

The aim of this article is to provide an overview of the disturbances that may affect the developing dentition. The focus is on the key anomalies that dentists should look out for during routine examination of children and adolescents and the possible adverse sequelae due to a delayed diagnosis. This will help ensure timely referral of patients to specialist services for early management.

## Key points

- An update for general dental practitioners on what to look out for during routine dental check-ups for children.
- Provides a detailed look into assessing for incisor and canine eruption and ensuring appropriate referral for treatment when necessary.
- Uses clinical cases to demonstrate possible sequelae of delayed referral, both positive and adverse.
- Abstract

## Introduction

General dental practitioners (GDPs) are in an ideal position to identify any anomalies in the developing dentition that can be addressed early and contribute to the development of a stable, aesthetic and functional occlusion. Late referrals can result in delayed and more complex treatment that can have adverse sequalae. Most commonly, ectopic permanent canines when referred late or when there has been a failure in interception may lead to root resorption, which can affect around 47% of adjacent teeth.^1^ Delayed referral can also result in surgical or more complex treatment plans, which can be avoided with early intervention.^2^ Dental Protection respond to numerous cases every year regarding delays in diagnosis and subsequently referral of ectopic permanent canines.^3^ Audits from orthodontic units around the United Kingdom demonstrate that impacted canines are often referred late, beyond the age of 12, in 50–76% of cases.^4^^,^^5^ GDPs have a responsibility to identify any abnormalities in the developing dentition and appropriately refer and/or intercept, depending on their level of experience and competence as per the General Dental Council Standard 7.1.^6^

The British Orthodontic Society (BOS) (2023)^7^ and American Academy of Paediatric Dentistry (2024)^8^ have published guidance on the management of the developing dentition and occlusion in paediatric patients.

**Fig. 1:**
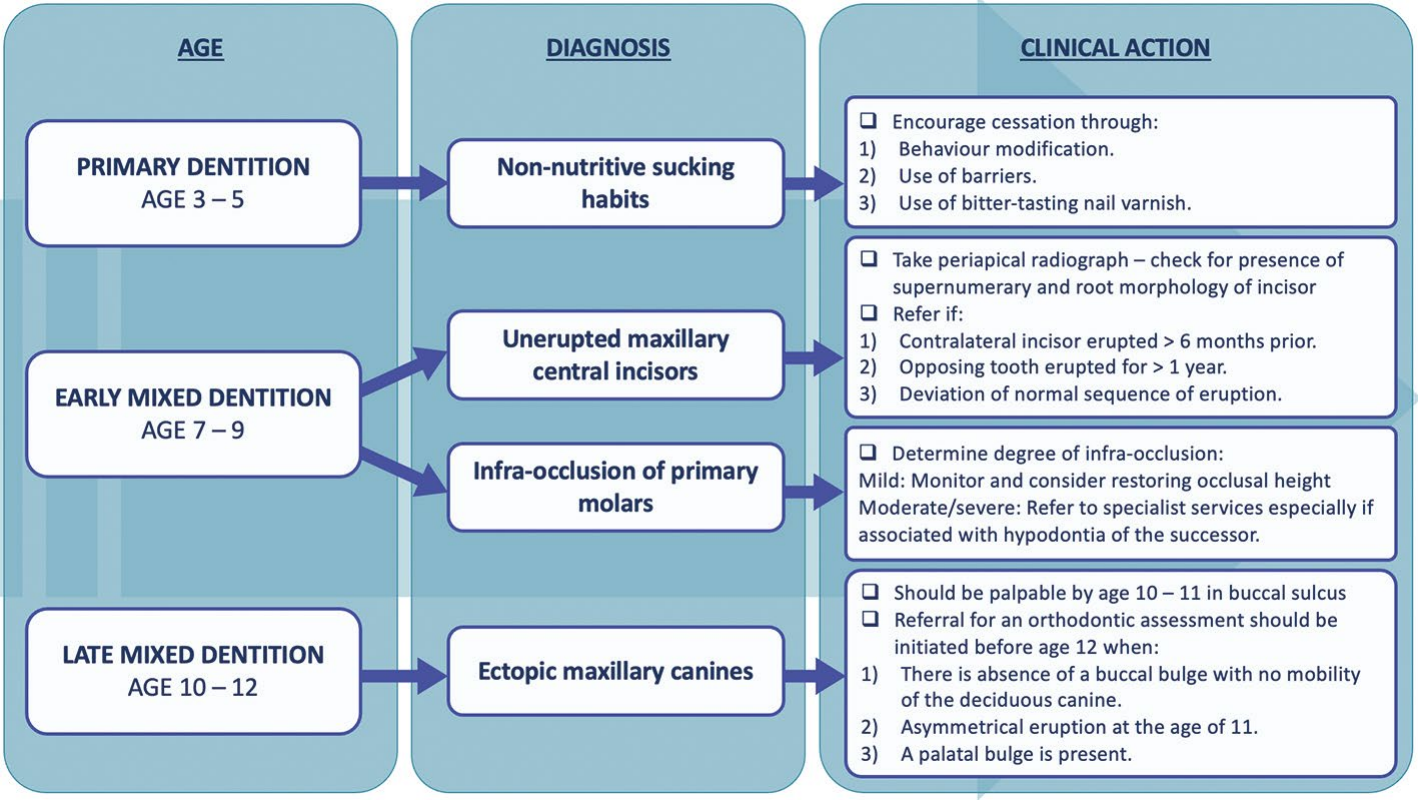
Summary of the key anomalies that can occur during the different stages of the developing dentition

The focus of this article is to provide guidance for GDPs on the key common anomalies to look out for during various stages of the developing dentition to ensure timely and appropriate referral and avoid possible adverse sequalae (Fig. 1).

## Primary dentition (3–5 years)

### Non-nutritive sucking habits

Non-nutritious sucking, including the use of dummies/pacifiers and digit sucking persisting for more than six hours a day, may affect the developing dentition.^9^ There is often a psychological element with children using this habit as a self-soothing mechanism which can make management more challenging. Most children grow out of the habit by the age of three years old, but for others, it persists and can be linked to stress and sadness.^10^ A study undertaken in Kettering in 2008 found 12.1% of children reported a digit-sucking habit past the age of seven years old.^11^ The most likely features of the malocclusion are an anterior open bite, a unilateral posterior crossbite, maxillary incisor proclination, and retroclination of the mandibular incisors resulting in an increased overjet.^12^

**Fig. 2:**
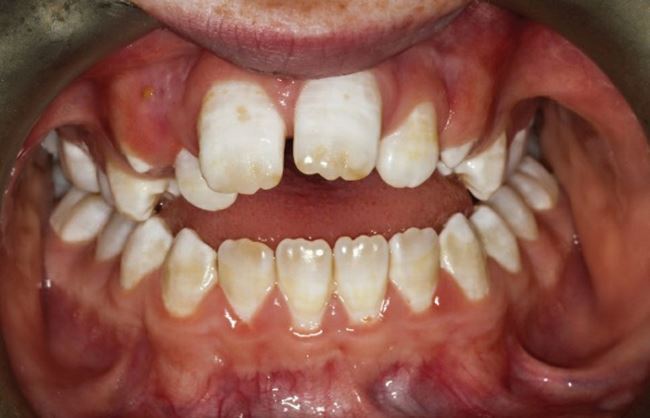
Features of the malocclusion seen in a 12-year-old child with a persistent digit-sucking habit

GDPs are well-positioned during routine dental visits to enquire about these habits and recognise clinical signs during development of the dentition. Figure 2 demonstrates the clinical presentation of a 13-year-old child who had a persistent digit-sucking habit until the age of 12 years old. They presented with a severe anterior open bite measuring a maximum of 6 mm with failure of development of the maxillary dental alveolus anteriorly. The case is further complicated by a diagnosis of hypomature amelogenesis imperfecta. The patient is planned for combined orthodontics and orthognathic surgery at the end of growth to address the anterior open bite.

Management interventions are outlined in Table 1. These techniques should be employed for a minimum of six months.^10^^,^^13^ A combination of approaches is often necessary, including behaviour modification and psychological support.^14^ Physical barriers have also been designed to assist with habit cessation, such as Thumbsie.^15^ Orthodontic habit breakers can also be used;^16^ however, a Cochrane review highlighted the weak evidence available with regards to interventions for non-nutritive sucking habits. The review analysed a number of various treatment modalities, including psychological treatments, primarily positive reinforcement, orthodontic appliances and aversive tasting substances. The results of this review found that no treatment modality was superior to another.^9^ The BOS also has further guidance on the management of non-nutritive sucking habits included in the *Managing the Developing Occlusion* document.^7^ Habits persisting into the permanent dentition can cause irreversible changes which can often only be treated appropriately with complex orthodontics and potentially orthognathic surgery with varying levels of long-term stability.^17^ Therefore, addressing these habits early may allow for self-correction and avoid the need for complex treatment.

**Table 1:** Management strategies for non-nutritive sucking habits

**Age**	**Suitable interventions**
Less than 2 years old	Introduce orthodontic dummy if digit-sucking persists^10^
3–7 years old and digit sucking	Psychological therapy including positive reinforcement, habit reversal and differential reinforcement of other behaviours^14^ Barrier method including gloves/plasters or specifically marketed products providing a glove-like barrier, for example, Thumbsie^15^ Bitter-tasting nail varnish
7–12 years old	May be suitable for removable or fixed orthodontic habit breaker including a bluegrass appliance^16^

## Early mixed dentition (7–9 years)

### Unerupted central maxillary incisors

The eruption of maxillary incisors should be monitored by GDPs, with most erupting around 7–7.5 years of age.^18^ They are the third most impacted tooth, with a higher incidence in boys compared to girls.^19^^,^^20^ Unerupted maxillary incisors can be regarded as unattractive and have a potentially negative influence on facial and dental aesthetics, which may impact upon self-esteem and social interaction of affected children.^21^ Failure of eruption is commonly associated with the presence of a supernumerary (most commonly, a tuberculate supernumerary present palatally), ectopic position, and trauma to the primary dentition, which may result in dilaceration of the permanent central incisor.

The Royal College of Surgeons of England have published guidelines on the management of unerupted central incisors.^22^

During routine examination of a child between the age of 6–9, clinical suspicion should be raised if:

- The contralateral incisor has erupted more than six months prior
- The opposing tooth has been erupted for over a year
- There is a deviation of normal sequence of eruption.

Other important considerations include retention of the primary incisor, lack of space (9 mm should be available) due to rotations, or drift of the lateral incisor into the central incisor space. The angulation and inclination of adjacent teeth should also be noted, as well as the presence of labial and palatal swellings.^18^

**Fig. 3:**
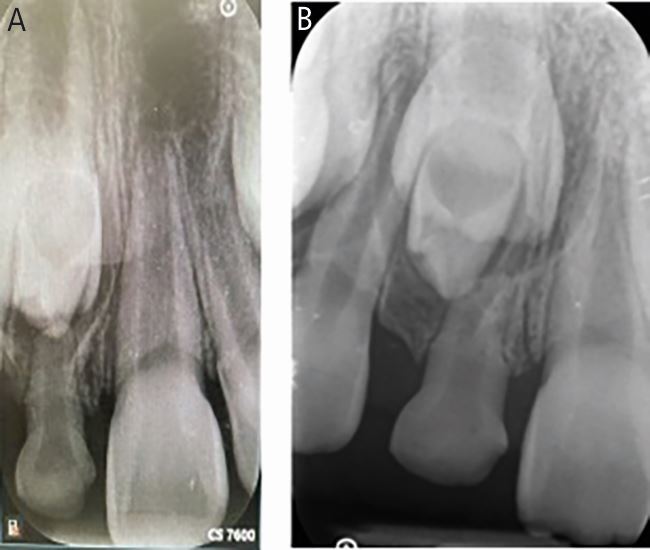
(A, B) Ten-year-old child referred for an impacted central incisor with a retained primary central incisor with an associated tuberculate supernumerary. Using parallax with two periapical radiographs the supernumerary was confirmed to be palatal

Radiographs are indicated if a delayed or unusual eruption pattern is noted. Use of parallax with two radiographs at different angulation can supplement determining the position of the incisor, as well as any obstruction (Fig. 3). This is based on Clark's buccal object rule with palatally positioned objects moving in the same direction of the beam.^23^ These can include horizontal parallax using periapical radiographs or vertical parallax using a periapical and upper standard occlusal, or a panoramic radiograph and an upper standard occlusal. Cone beam computed tomography (CBCT) can also be useful in selected cases where exposure is justified.^18^

When a GDP suspects an unerupted central incisor, prompt referral to their local orthodontic unit is justified. Earlier referral results in better outcomes due to the eruptive potential of the tooth with an immature apex, as well as to avoid space loss with tipping of the adjacent teeth.^24^ Furthermore, there is a likely negative psychosocial impact on children, particularly if treatment is delayed until they enter secondary school when bullying is more prevalent between the ages of 12–14.^21^

Various factors are considered in the management of the impacted central incisor, outlined in Table 2.

**Table 2:** Factors considered during treatment planning for impacted incisors

**Patient factors**	**Dental factors**
Medical history	Position of the impacted incisor particularly vertical height. A higher vertical position has been associated with a lower chance of spontaneous eruption after removal of a supernumerary^16^
Cooperation and compliance with varying treatment modalities	Nature of the physical obstruction (such as the morphology and location of the supernumerary)^16^
Patient and parental preference	Unfavourable root formation (such as dilaceration).^17^ Chaushu *et al.* (2015) reported a high risk of failure with dilacerated incisors^16^
Age of the patient (correlating to root development stage)	Stage of root development^16^

Management includes surgical removal of the physical obstruction alone, or removing the obstruction and creating space for eruption orthodontically with surgical exposure of the unerupted incisor.^24^ When surgical removal of the obstruction is being arranged under general anaesthetic, concurrent exposure and bonding of an orthodontic attachment is best practice to avoid repeat general anaesthetic.^25^ It is essential that a robust and comprehensive treatment plan is made before the general anaesthetic, as highlighted in the recent *Getting It Right First Time* hospital dentistry report by NHS England.^26^ This will usually involve an orthodontic/paediatric dentistry assessment before the general anaesthetic. Removal of the incisor may be indicated in cases of significant ankylosis or dilaceration. Space should be maintained for the eventual placement of a fixed or removable replacement prosthesis.^18^ Autotransplantation is another option in which another developing tooth, such as a premolar, is used as a replacement for an unerupted central incisor.

Importantly, following treatment of impacted maxillary incisors, affected children should be monitored closely by their GDP as 41% will likely have an impacted canine on the ipsilateral side compared to 4.7% on the contralateral side.^27^

**Fig. 4:**
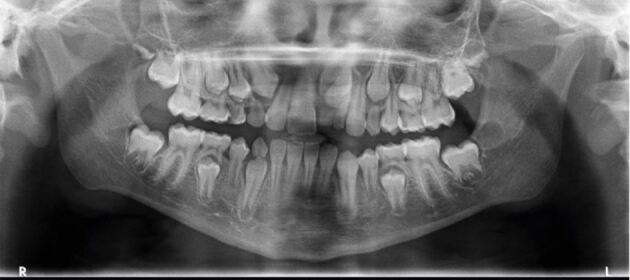
Panoramic radiograph taken for a ten-year-old child to assess the overall development of the dentition, as well as the unerupted upper left central incisor

**Fig. 5:**
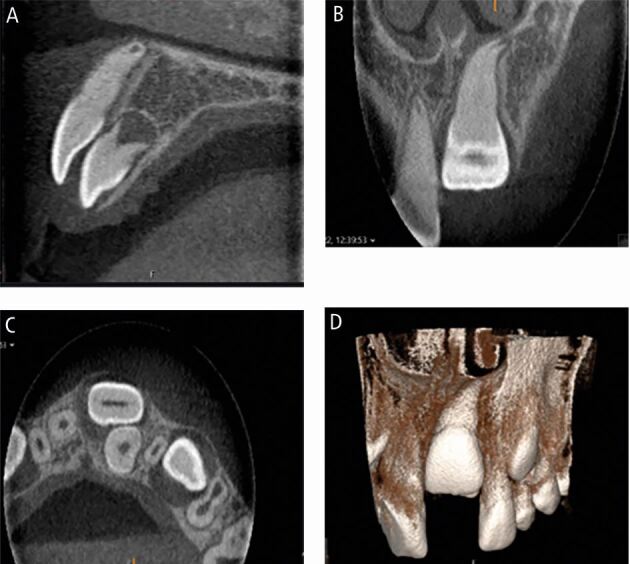
(A) Sagittal view of small-volume cone beam computed tomography (CBCT) confirming palatally positioned supernumerary tooth. (B) Coronal view of CBCT demonstrating apical dilaceration of the upper left central incisor root and close relationship with the nasal floor. (C) Axial view confirming palatal supernumerary. (D) 3D reconstruction of unerupted left central incisor

We hereby discuss a case of late referral of an impacted central incisor to the orthodontic department in a district general hospital. A ten-year-old child was referred with retained upper left primary incisors and an unerupted left maxillary incisor. The upper left lateral incisor had erupted distal to the upper left lateral primary incisor. The patient had previous dental trauma at four years old with an intrusion injury to the upper left primary central incisor. An initial panoramic radiograph confirmed the upper left impacted central incisor with the presence of a supernumerary tooth (Fig. 4). Due to the history of trauma and failure to visualise the roots of the upper left central incisor on the panoramic radiograph, the patient was referred for a CBCT scan (Fig. 5). The patient had extraction of the retained upper left primary incisors under local anaesthetic before the CBCT scan. The scan confirmed a tuberculate supernumerary palatal to the central incisor with a mild apical dilaceration of the central incisor root which was intimately related to the nasal floor.

Following extraction of the supernumerary and exposure and bonding of a gold chain attachment to the central incisor carried out under a general anaesthetic, tooth 21 erupted spontaneously and the gold chain was removed with orthodontic treatment no longer necessary. Patients with a history of dental trauma (especially intrusion injury in the primary dentition) should be monitored closely as this can cause disturbance to the developing permanent tooth germ, as well as root dilaceration, which increases the risk of impaction.^28^ This reduces the surgical and treatment burden for these young children if treatment is initiated earlier.

### Infraocclusion of primary molars

Primary teeth can become ankylosed and infraoccluded whereby they fail to maintain their vertical position in relation to the occlusal plane, with an incidence of 8–14%.^29^ Failure to detect infraocclusion can result in complete infraocclusion below the mucosa and subsequent surgical extraction, resulting in vertical bony defects with difficulty restoring the edentulous area in the future.^30^ GDPs should aim to spot any signs of infraocclusion and address/refer if appropriate.

Infraoccluded teeth are normally ankylosed, confirmed occasionally by the presence of the metallic sound on percussion. There can be potential space loss, tipping of adjacent teeth and overeruption of opposing teeth, as well as centreline shifts and lateral open bites.

Messer and Cline (1980) classified infraocclusion as mild, moderate and severe. Mild infraocclusion is whereby the occlusal surface is less than 2 mm below the adjacent teeth, moderate the occlusal surface is at the level of the interproximal contact, and in severe infraocclusion, the occlusal height is below the interproximal contact.^31^ Infraoccluded primary teeth are not amenable to orthodontic treatment. Treatment options depend on the severity of the infraocclusion, the age of the patient, and presence or absence of the successional premolar, as well as other features of the malocclusion. A recent retrospective study looking at the surgical complexity of dental implant surgery for infraoccluded teeth with hypodontia of the permanent successor found maintenance of the retained primary molar may preserve buccolingual bone volume for future implant cases. However, a thorough patient-specific assessment should be made regarding the decision to extract or maintain in cases of hypodontia of the successor, taking into account the factors mentioned above with referral to a local specialist orthodontic or paediatric dentistry service.^32^

Maintaining the tooth is often indicated if:

- There is hypodontia of the premolar
- There is good root length and coronal tooth structure remaining
- The infraocclusion is not judged to be severe with risk of submergence below the mucosa, disturbance to the occlusion, or risk of food packing with resulting caries and periodontal disease.

Restoration of occlusal height may also be performed to prevent tipping, overeruption and crowding of adjacent teeth, especially if there is hypodontia of the premolar.^30^^,^^33^ This can be performed in primary care using composite or alternatively preformed metal crowns.

Extraction may be indicated in cases if:

- There is a risk of future submergence, especially if the infra-occlusion is noted before the pubertal growth spurt, as it is likely to worsen with growth, making extraction difficult and increasing the risk of a vertical bony defect. If the tooth is already 2–3 mm below the occlusal level before the growth spurt, early extraction is advised
- The premolar is showing signs of a disturbed eruptive path with often distal tipping under the primary molar
- The premolar has more than half to two-thirds of the root formed and the primary molar is not mobile.

**Fig. 6:**
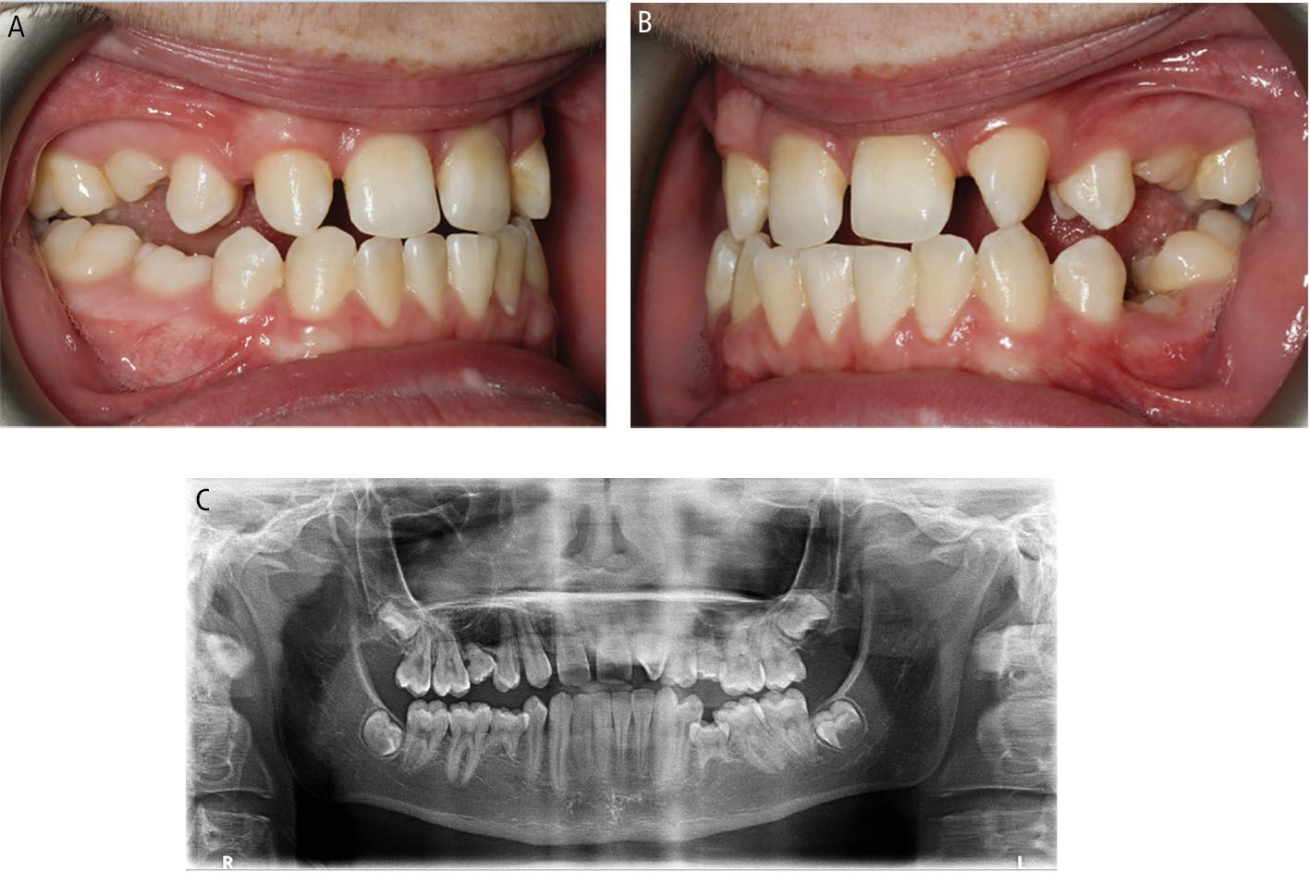
(A, B) Clinical photographs demonstrating severe infraocclusion affecting the upper right, upper left and lower left second primary molars with moderate infraocclusion of the lower right second primary molar. Tipping of the adjacent teeth was noted with lateral open bites posteriorly between the infraoccluded teeth. (C) Panoramic radiograph confirming the clinical findings and minimal root lengths remaining on the retained primary molars

Figure 6 demonstrates a case whereby a 15-year-old patient was referred with a Class III malocclusion with infraoccluded second primary molars associated with hypodontia of the second premolars and the upper lateral incisors. The patient was treated with extraction of the primary molars and space closure in the lower arch, with space redistribution for restoration of the upper lateral incisors with resin-bonded bridges.

In summary, GDPs are well-positioned to identify and investigate infraocclusion of primary molars and should liaise/refer appropriately to orthodontic/paediatric dental colleagues for management of complex cases.

## Late mixed dentition (10–12 years)

### Ectopic maxillary canines

Maxillary permanent canines are the second most commonly impacted teeth.^34^ The incidence ranges from 0.27–2.4%,^35^^,^^36^ to 1.1% in a Swedish population, where interceptive treatment is systematically implemented.^37^ Girls are 2.3–3 times more likely to be affected than boys.^38^ Brorsson and Naoumova (2020), in their recent prospective longitudinal study using CBCT, found 75% of maxillary impacted canines to be palatal.^39^ This high frequency of palatal impaction is attributed to buccal canines often erupting buccally but displaced due to crowding. Failure to diagnose an ectopic maxillary canine early can result in lengthy orthodontic treatment, as well as potential resorption of adjacent teeth.^1^^,^^40^

Eruption of canines should be monitored by the GDP during the late mixed dentition. Canines with normal eruption path should be palpable in the buccal sulcus by 10–11 years of age and erupt at the mean age of 10.8 in girls and 11.6 years old in boys, with 3–4 years of individual variation.^41^ The key features for clinical assessment of canine development are outlined in Table 3.

**Table 3:** Clinical assessment of maxillary canine development and eruption

**Clinical assessment**	**Features**
Visual inspection	Delayed eruption of the canine Retention of the deciduous canine with lack of mobility Asymmetry of dental development Inclination and angulation of the lateral incisor, which may indicate impingement of an impacted canine on its root^33^ Children with dental anomalies have a 2.5-fold increased risk of developing an impacted canine compared to those without dental anomalies.^34^ The anomalies include microdont lateral incisors, hypodontia of any tooth or impaction of other teeth^35^
Palpation	Commenced from the age of ten with 70% of canines palpable, increasing to 95% by 11 years of age. A palatal bulge may be palpable if the tooth is palatally impacted^36^

Radiographic assessment is indicated if the canine is not palpable to ascertain its position.^42^ This may involve the use of two radiographs at different angulations for parallax. A panoramic radiograph is often the first radiograph of choice. Traditionally, an upper standard occlusal radiograph was used for vertical parallax; however, due to the increased diagnostic accuracy of a CBCT to detect root resorption, it may be prudent to proceed directly to a CBCT if clinically indicated following the panoramic radiograph considering the cumulative doses are comparable. Therefore, it may be best for a GDP to consider only taking a panoramic radiograph and allow the specialist service to determine if further imaging is required.^43^

National clinical guidelines published by the BOS and the Royal College of Surgeons of England recommend early palpation of canines to avoid a delay in diagnosis.^44^ Referral for an orthodontic assessment should be initiated before the age of 12 when there is:

- Absence of a buccal bulge with no mobility of the primary canine
- Symmetric eruption at the age of 11
- Palatal bulge.

Patel *et al.* (2016) found that 76% of patients referred to the Royal Surrey County Hospital were referred late (>12 years old) at a mean age of 14.1 years.^5^ GDPs have a duty to monitor the development of the dentition and identification of the position of maxillary permanent canines should routinely form part of the dental examination.

Once referral has been initiated to an orthodontist, the main treatment options include:

1. Interceptive extraction of the primary canine
2. Exposure and alignment of the canine with fixed appliances
3. Extraction of the impacted canine
4. Autotransplantation.

**Fig. 7:**
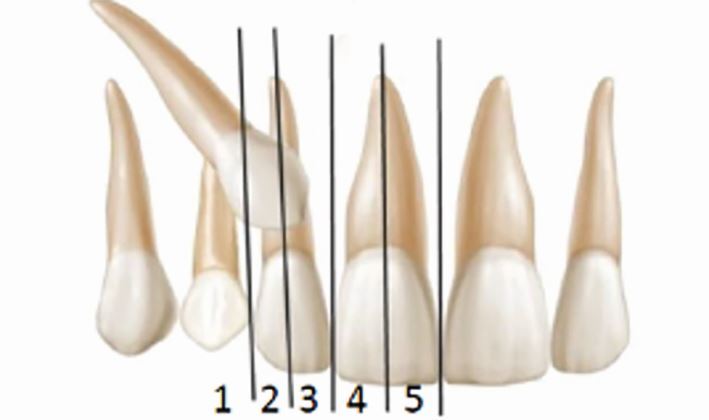
Classification of position of impacted canines by sector position

**Fig. 8:**
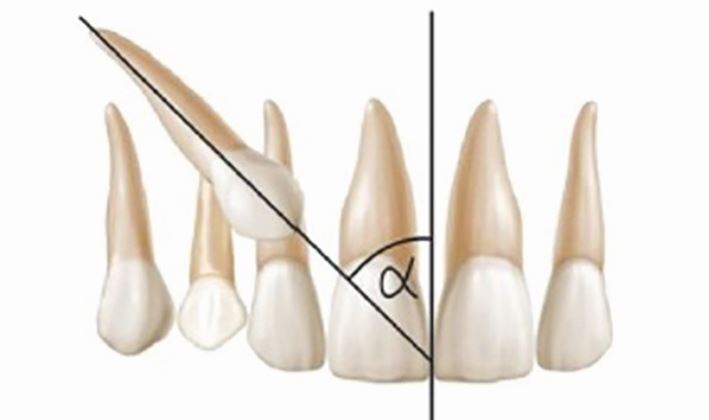
Alpha angle of impacted maxillary canine: angle between long axis of the canine and the midline^42^

Interceptive extraction of the primary canine is best carried out for canines with a sector position of one, two and three (Fig. 7).^42^ Successful eruption has been noted for cases with an alpha angle up to 45 degrees with reduced success with an angle greater than 31 degrees (Fig. 8). The updated Royal College of Surgeons of England guidelines in 2022 outline that interceptive extraction can improve the position of palatally displaced canines in carefully selected patients when the canine is not severely displaced and has other favourable criteria, such as an uncrowded arch and age range of 10–13 years old. Alternative treatment should be sought if there is no improvement by 12 months following the intervention.^44^

Early referral allows for interceptive treatment or exposure and alignment if necessary. Failure to diagnosis or treat an ectopic canine can result in numerous sequalae. These include root resorption of adjacent teeth reported to affect 38% of lateral incisors and 9% of central incisors.^1^ Failure to treat at an earlier stage may also result in worsening of the ectopic position leading to an increased treatment time. The average treatment time for impacted canines is 26.3 months.^40^ Success rates for both interceptive treatment and alignment also decrease with age. Naomova *et al.* (2015) found interceptive extraction to be more successful in the younger age group (10–11 years old) in their randomised controlled trial compared to the 12–13-year-old group.^2^ Becker and Chaushu (2003) found success rates for orthodontic alignment of canines in adults (20–47 years) to be 69.5% compared to 100% in a younger cohort (12–16 years old).^45^ Due to the complexity and multi-disciplinary nature of the treatment, often requiring a general anaesthetic, alignment of a canine is a substantial burden on NHS (National Health Service) resources.

**Fig. 9:**
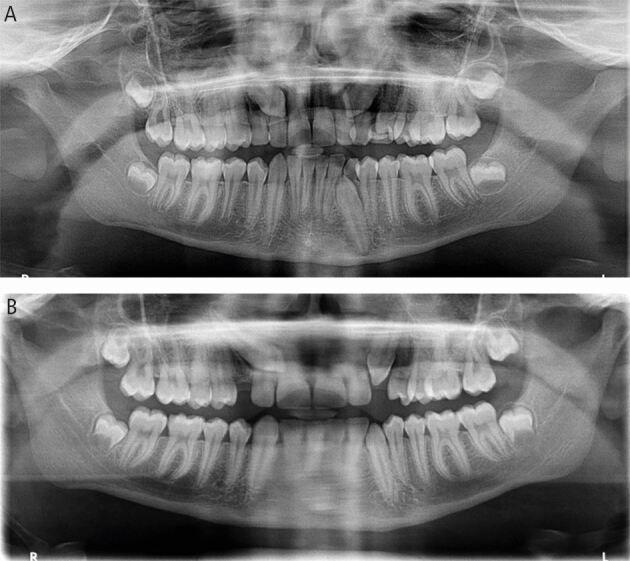
(A, B) Pre- and post-interceptive extraction panoramic radiographs for a 12-year-old patient where extraction of the upper and lower left primary canines was carried out

We herein discuss a case of timely referral for interceptive extraction of impacted maxillary canines in a 12-year-old referred by their GDP when the primary canines were not mobile and the maxillary canines not palpable buccally (Fig. 9). The patient also had an impacted lower left canine. The patient met the appropriate criteria for interceptive primary canine extraction with well-aligned arches. The extractions of the primary canines were carried out by the GDP and on review one year later, the impacted canines on the left side had erupted. Unfortunately, the upper right canine failed to respond, with a worsening of the sector position to four with an alpha angle of 45 degrees. This case demonstrates the findings of the best available evidence. Naoumova *et al.* (2015) reported success rates of 69% in the interceptive extraction of the primary canine group compared to 39% in the control group as the outcome from their randomised controlled trial.^2^ Although, they found favourable results for canines with a sector three position, the alpha angle of 13 in this case was 40 degrees. According to their most recent study, they recommend exposure and orthodontic alignment of impacted canines for cases when the alpha angle is greater than 30 degrees as an increased alpha angle is associated with reduced success rates.^46^

**Fig. 10:**
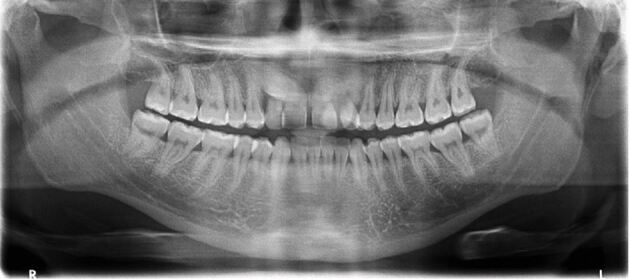
A panoramic radiograph of a 26-year-old patient with an impacted upper right canine and palatally displaced upper left canine

**Fig. 11:**
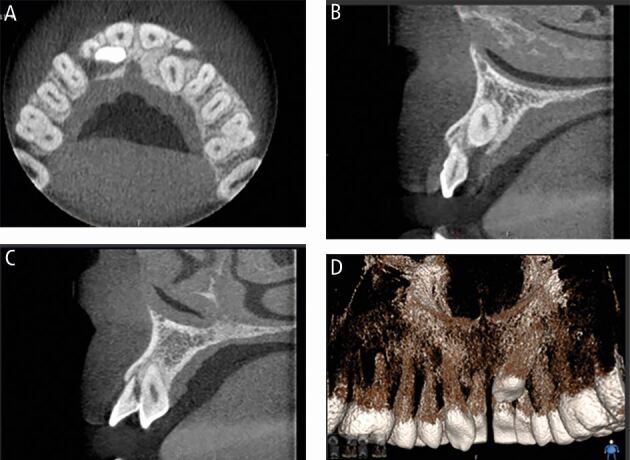
(A) Coronal view of the impacted upper right canine with severe resorption of 12 and moderate resorption of 11. (B) Sagittal view of palatally impacted upper right canine with severe resorption of the upper right lateral incisor. (C) Sagittal view of upper left canine demonstrating severe resorption of the upper left lateral incisor with 3 mm of remaining root support in bone. (D) 3D reconstruction from the palatal aspect of the maxillary arch

Late referral can have sequalae that are demonstrated in the case below. A 26-year-old patient with a Class III malocclusion was referred by their GDP with an unerupted 13 and palatally displaced 23. The patient was unaware of the impacted canines until the 23 began to erupt palatal. There was severe crowding in the upper arch with insufficient space for the canines. The upper lateral incisors were also grade two mobile. A panoramic radiograph confirmed the impacted upper right canine (Fig. 10). The upper left canine was magnified due to its palatal position and difficult to visualise on the radiograph. The roots of the upper lateral incisors were not fully visible but the upper right lateral incisor root appeared to have severe resorption into the pulp. A CBCT was taken confirming severe resorption of the upper lateral incisors, as well as evidence of possible ankylosis around 13 and 23 (Fig. 11). The patient underwent extraction of the impacted 13, severely resorbed and poor prognosis 22, and the lower first premolars to correct the malocclusion. The upper right lateral incisor was accepted in its position. This tooth has severe resorption and the patient was warned this tooth is likely to be lost in the future with need for prosthetic replacement. This case highlights the sequalae of delayed diagnosis and referral of impacted canines.

## Conclusion

GDPs should be aware of the possible anomalies which can present during the development of the dentition and understand when to refer. Eruption of the teeth should be monitored closely during the developing dentition, particularly central incisors and maxillary canines, with timely referral if any disturbance is noted. Late diagnosis and referral can result in numerous irreversible sequalae.
